# Comparative genomic identification and validation of β-defensin genes in the *Ovis aries* genome

**DOI:** 10.1186/s12864-017-3666-x

**Published:** 2017-04-04

**Authors:** T. J. Hall, C. McQuillan, E. K. Finlay, C. O’Farrelly, S. Fair, K. G. Meade

**Affiliations:** 1 Animal & Bioscience Research Department, Animal & Grassland Research and Innovation Centre, Teagasc, Grange, Dunsany, Co Meath, Ireland; 2 Comparative Immunology Group, School of Biochemistry and Immunology, Trinity Biomedical Sciences Institute, Trinity College Dublin, Dublin 2, Ireland; 3 Laboratory of Animal Reproduction, School of Natural Sciences, Faculty of Science and Engineering, University of Limerick, Limerick, Ireland

**Keywords:** Ram, Epididymis, Sheep, Fertility

## Abstract

**Background:**

β-defensins are small, cationic, antimicrobial peptides found in species across the plant and animal kingdoms. In addition to microbiocidal activity, roles in immunity as well as reproduction have more recently been documented. β-defensin genes in *Ovis aries* (domestic sheep) have been poorly annotated, having been identified only by automatic gene prediction algorithms. The objective of this study was to use a comparative genomics approach to identify and characterise the β-defensin gene repertoire in sheep using the bovine genome as the primary reference.

**Results:**

All 57 currently predicted bovine β-defensin genes were used to find orthologous sequences in the most recent version of the sheep genome (OAR v4.0). Forty three genes were found to have close genomic matches (>70% similarity) between sheep and cattle. The orthologous genes were located in four clusters across the genome, with 4 genes on chromosome 2, 19 genes on chromosome 13, 5 genes on chromosome 20 and 15 genes on chromosome 26. Conserved gene order for the β-defensin genes was apparent in the two smaller clusters, although gene order was reversed on chromosome 2, suggesting an inversion between sheep and cattle. Complete conservation of gene order was also observed for chromosome 13 β-defensin orthologs. More structural differences were apparent between chromosome 26 genes and the orthologous region in the bovine reference genome, which is known to be copy-number variable. In this cluster, the Defensin-beta 1 (*DEFB1*) gene matched to eleven Bovine Neutrophil beta-Defensin (BNBD) genes on chromosome 27 with almost uniform similarity, as well as to tracheal, enteric and lingual anti-microbial peptides (*TAP*, *EAP* and *LAP*), suggesting that annotation of the bovine reference sequence is still incomplete. qPCR was used to profile the expression of 34 β-defensin genes, representing each of the four clusters, in the ram reproductive tract. Distinct site-specific and differential expression profiles were detected across the reproductive tract of mature rams with preferential β-defensin gene expression in the epididymis, recapitulating observations for orthologous genes in other species.

**Conclusions:**

This is the first comprehensive analysis of β-defensin genes encoded by the ovine reference sequence, and the first report of an expanded repertoire of β-defensin genes in this species. The preferential expression of these genes in the epididymis suggests a role in fertility, possibly providing immunoprotection for sperm within the female reproductive tract.

**Electronic supplementary material:**

The online version of this article (doi:10.1186/s12864-017-3666-x) contains supplementary material, which is available to authorized users.

## Background

Defensins are small cationic peptides with a characteristic conserved six cysteine signature and are present in a wide range of species from insects to plants and animals. On the basis of differences in their size and their cysteine residue spacing, mammalian defensins are divided into α, β, and θ sub-classes [1]. α-defensins are characterised by having disulphide bonds between cysteines in positions 1–6, 2–4 and 3–5 whereas the cysteine disulphide bonds of β-defensins lie between 1 and 5, 2 and 4 and 3 and 6 [2]. The third type, θ-defensins are a recently acquired primate specific class of peptide, which are formed by the merging of the other two class of defensins [3]. The sub category β-defensins are most numerous among vertebrates and have been extensively studied. Their usual gene structure consists of 2 exons, the first exon containing the 5’ untranslated region and signal peptide of the preproprotein; the second encoding the mature peptide with the characteristic conserved six cysteine domain [4].

β-defensins are produced by phagocytic cells, leukocytes and epithelial cells within the gastrointestinal tract, liver, skin and lungs [5] and constitute an important and versatile component of the innate immune system [6]. Originally described as anti-microbial peptides (AMPs) because of their microbicidal activity [4, 7], additional immunoregulatory and stimulatory functions has led to the term host defence peptide [8, 9]. It is now known that β-defensins link the innate and adaptive immune responses in higher organisms, by acting as signalling molecules in the immune system and chemoattractants for T-lymphocytes and immature dendritic cells [10]. More recently a role in fertility has been also demonstrated for these pleiotropic molecules [11].

Many studies have documented expression of β-defensin genes along the epididymis, particularly in the caudal region of the epididymis [12, 13]. The epididymis is a single, convoluted duct, which can reach about 50 m in length in sheep and up to 80 m in horses. Attached along the caudomedial border of the testis, the epididymis can be divided into three main segments, the caput (head), corpus (body) and cauda (tail), which finally tapers into the vas deferens. It is through this tube that sperm progressively acquire functional competency for fertilisation, and therefore characteristic highly regionalised gene and protein expression profiles give rise to a dynamic intraluminal environment [14]. One particular β-defensin, (BD126) has been documented to play a number of roles critical to sperm survival, motility and interaction with the female reproductive tract and its secretions [15–17]. A dinucleotide deletion in the human BD126 gene has also been associated with subfertility in men [18]. In agreement with their broad role in reproductive physiology, a recent study has also shown that β-defensin gene knock-out male mice are infertile [19].

Extensive analysis of β-defensin gene expression profiles have been performed in humans and model-organisms, the same is not true for the majority of livestock species. Our group discovered a cluster of novel β-defensin genes in the bovine genome [20] which were preferentially expressed in the reproductive tract in the bull [21]. Immunohistochemistry and Western-blotting showed that bovine BD126 (bBD126) exists as a dimer expressed by epithelial cells of the caudal epididymis and vas deferens and coats sperm [22]. Furthermore, addition of the recombinant bBD126 protein induces increased motility of immature corpus sperm [23].

To date, only two β-defensin genes have been characterised in sheep in any detail. Originally discovered in 1998, sheep β-defensin 1 (*sBD1*, also referred to as *oBD1*) and 2 (*sBD2*/*oBD2*) were identified in the trachea and ileum, respectively [24, 25]. *oBD2* mRNA and peptide expression were highest in the intestinal tract and tissue distribution progressively decreases with maturity [26]. The induction of these AMPs in response to viral and bacterial infection suggests they may play a role in mediating disease resistance [27, 28], however their expression in reproductive tissues has not previously been assessed.

Here, we used a comparative genomics approach to identify and classify β-defensin genes in the ovine reference genome. Based on our work in related species [21, 29], expression profiling was used to validate these predicted sequences and provide insight into a potential functional role for these novel genes.

## Methods

### Bioinformatic identification of ovine β-defensin orthologs

All 57 characterised bovine β-defensin genes [20] were used to identify orthologs in the *Ovis aries* genome (OAR v4.0) via the basic local alignment search tool (BLAST) [30] using TBLASTN, which compares the query peptide sequence to the subject DNA translated in all six open reading frames. Only sequences exceeding 70% identity and 50% coverage were selected for further study. The 43 predicted *Ovis aries* β-defensin protein sequences were aligned using Clustal Omega [31] and Jalview [32]. Identified ovine β-defensin peptide sequences were also searched against the UniProt and Ensembl database to identify any β-defensin homologs not found using the NCBI database reference genome. Chromosomal locations and exon/intron boundaries were determined by BLAST-like alignment tool (BLAT [33]) and reconfirmed via GenScan [34].

BLAST retrieved ovine β-defensin sequences often had to be manually completed before phylogenetic analysis could be carried out properly, due to short sequence length and sequence diversity of the β-defensins. Missing first exons for *oBD128*, *oBD126*, *oBD117*, *oBD121*, *oBD108* and *oBD109a* were discovered by extracting a section of the sheep reference genome via NCBI genome browser that lay between the defensin of interest and the nearest upstream β-defensin. These chromosomal intervals were then translated via Linux based EMBOSS transeq into all six open reading frames. The bovine first exons most closely related to the ovine β-defensin gene of interest were aligned to this translation to find a potential first exon. The new first exons and their respective second exons were then aligned to the entire bovine and sheep β-defensin repertoire to determine similarity. First exons for *oBD104*, *SPAG11E* and *oBD1* could not be found using this method, as the alignment of the bovine first exons to the region of interest proved inconclusive due to low similarity and coverage.

Hidden Markov Models (HMM) searches of the ovine reference sequence were also carried out to identify novel β-defensin sequences. To achieve this, genome intervals in which the β-defensin clusters lay were translated into all six open reading frames using the Linux based EMBOSS transeq [35]. The HMM was then built using a T-coffee alignment of the entire bovine and ovine characterised β-defensin repertoire, as well as the newly identified ovine β-defensin genes that exhibited high similarity to the bovine β-defensins, and the hmmbuild program in the HMMER 2.1.1 software package [36]. The nucleotide sequences were subsequently aligned using T-coffee [37] to determine putative domains between the cattle and sheep β-defensins.

Second exons were also evaluated and manually edited. Ovine genes *oBD125a*, *oBD125*, *oBD115*, *oBD118*, *oBD120*, *oBD109a*, *oBD128*, *oBD132* and *oBD131* did not have stop codons at the 3’ end of the second exons. A custom python script was written to extend all ovine defensins to the nearest in frame stop codon. The extended sequences were then aligned with the rest of the ovine β-defensins. To ensure that the extensions identified were not a result of poor genome assembly, sequences were then searched against the NCBI genome database to determine whether they existed in other organisms. With exception to *oBD1321* and *oBD132*, all extensions existed in 4 or more different species, including *Capra hircus* (goat), *Turisops truncates* (bottlenose dolphin) and *Canis lupus* (dog).

All ovine β-defensin gene and protein sequences (as well as genomic coordinates) are available in Additional file 1.

### Phylogenetic and syntenic analysis

β-defensin gene sequences with identified first and second exons were aligned via ClustalW2 with gap open and extension penalties of 7 and 0.2, respectively. The phylogenetic relationship between the β-defensin gene repertoire of cattle and sheep was then investigated via MEGA 5.1 [38], using the maximum likelihood method and a bootstrapping value of 100 for each tree constructed. Upon identifying both exon/intron boundaries and phylogeny, the conserved gene orientation between the different chromosomal clusters of β-defensins of both sheep and cattle was investigated and illustrated via ArkMAP [39].

### Reproductive tissue collection, RNA extraction and cDNA synthesis

Tissues were aseptically collected at a local abattoir from healthy rams (*n* = 5) within 30 min of slaughter. Reproductive tracts were retrieved from post pubertal 12 month old Suffolk rams and dissected to obtain 3 mm sized tissue samples of testis (T), caput (CT), corpus (CS) and cauda (CA) of the epididymis as well as the vas deferens (VD). Each tissue sample that was collected was preserved in RNAlater (Qiagen, Crawley, UK) and stored at -20 °C.

RNA was extracted using Trizol and RNeasy Plus Mini Kit with on-column DNA digestion (Qiagen Ltd, Crawley, UK). The quantity of the purified RNA was measured using NanoDrop ND-1000 UV–Vis Spectrophotometer (NanoDrop Technologies Inc., Wilmington, DE, USA). The quality of RNA was assessed on Agilent 2100 BioAnalyzer using Agilent RNA 6000 Nano Kit according to the manufacturer’s instructions (Agilent, Wharfedale Road, Wokingham, Berkshire, UK). RNA Integrity Numbers (RIN) >7 were obtained for all samples and the RNA stored at -80 °C. The High-Capacity cDNA Reverse Transcription Kit (Applied Biosystems, Warrington, UK) was used to convert 200 ng of RNA into cDNA as per manufacturer’s instructions. The reverse transcription process was carried out under the following conditions: 10 min at 25 °C, 120 min at 37 °C and 5 min at 85 °C. The stock cDNA was stored at -20 °C.

### Primer Design*, qPCR and data analysis*

Intron-spanning gene-specific primers were designed using Primer 3 (see Additional file 1). For *oBD122/oBD122a* and *oBD125/oBD125a*, primers were designed to amplify products from regions of exon 2 containing different nucleotides to maximise the chances of differentiating between the closely related sequences. Primer specificity was confirmed by Primer Blast (NCBI) before commercial synthesis (Sigma-Aldrich Ireland Ltd, Wicklow, Ireland).

Quantitative RT-PCR (qPCR) was performed on Applied Biosystems 7500 Fast Real-Time PCR System, using SYBR Green intercalating DNA dye, according to the manufacturer’s instructions (Applied Biosystems Ltd, Warrington, UK). The reaction efficiency for each primer pair was calculated using a two-fold dilution series on a pooled cDNA sample, obtained by pooling 2 μl of cDNA from each sample in a single eppendorf tube. The standard curves were represented as the semi-log regression line plot of Ct value vs. log of the relative input cDNA concentration. The efficiencies between 85% and 110% were considered acceptable and primers with efficiencies within these limits were included. All genes were normalised to the expression of *ACTB* as a reference gene. The software package GenEx 5.2.1.3 (MultiD Analyses, Gothenburg, Sweden) was used for qPCR data analyses [40]. Melt curve analysis was also performed to ensure a single product was formed and to ensure minimal formation of primer-dimer artefacts. Data obtained for oBD128 and oBD129 were excluded as expression levels were low in all tissues assessed and primer amplification excluded reliable quantification of product. The amount of target, normalised to an endogenous reference and relative to a calibrator, was: 2^-ΔΔCt^, where ΔCt is the difference in Ct between target and reference and ΔΔCt is the difference in ΔCt between all the samples and the calibrator, the sample with the lowest expression [41]. The differences in gene expression levels between tissue segments were analysed by ANOVA and Tukey post-hoc test as implemented in GraphPad Prism (Version 6).

## Results

### Prediction of β-defensin genes in the ovine reference genome

Bovine β-defensin genes, some of which were previously discovered by our group [20], were used to search the ovine reference genome to identify evolutionarily conserved orthologs. Of the 53 resulting hits, 41 passed the cut offs which included 50% sequence coverage, 70% sequence identity and an e-value of 10^-3 or lower. All of the resulting hits had at least 80% sequence identity, indicating a high likelihood of β-defensin homology. Additionally, the previously annotated *oBD1* and *oBD2* were also identified as having similarity with 14 β-defensins on chromosome 27, though the similarity was always between 67 and 69%. Of the 41 hits, *oBD128, oBD126, oBD117, oBD121, oBD104, oBD108, oBD109a* and *SPAG11E* did not have first exons identified via a BLAST hit. All first exons of the above genes, as well as the first exons for bovine defensins *BD117* and *BD108* were discovered after parsing through the genomic region the exons were predicted to lie within and making local alignments of first exons of genes that share high similarity with the missing first exons. The first exons of *oBD104* and *SPAG11E* however could not be identified using this method, as the first exons of the bovine ortholog could not be accurately aligned to any part of the sheep genome possibly due to incomplete sequence assembly. Additionally, *oBD128, oBD115, oBD119, oBD120, oBD122a, oBD113, oBD109a* and *oBD140* were found not to extend to their first in frame stop codon after analysis of the flanking genomic regions of the second exons. As such, each of these genes were extended to the first in frame termination site.

**Fig. 1:**
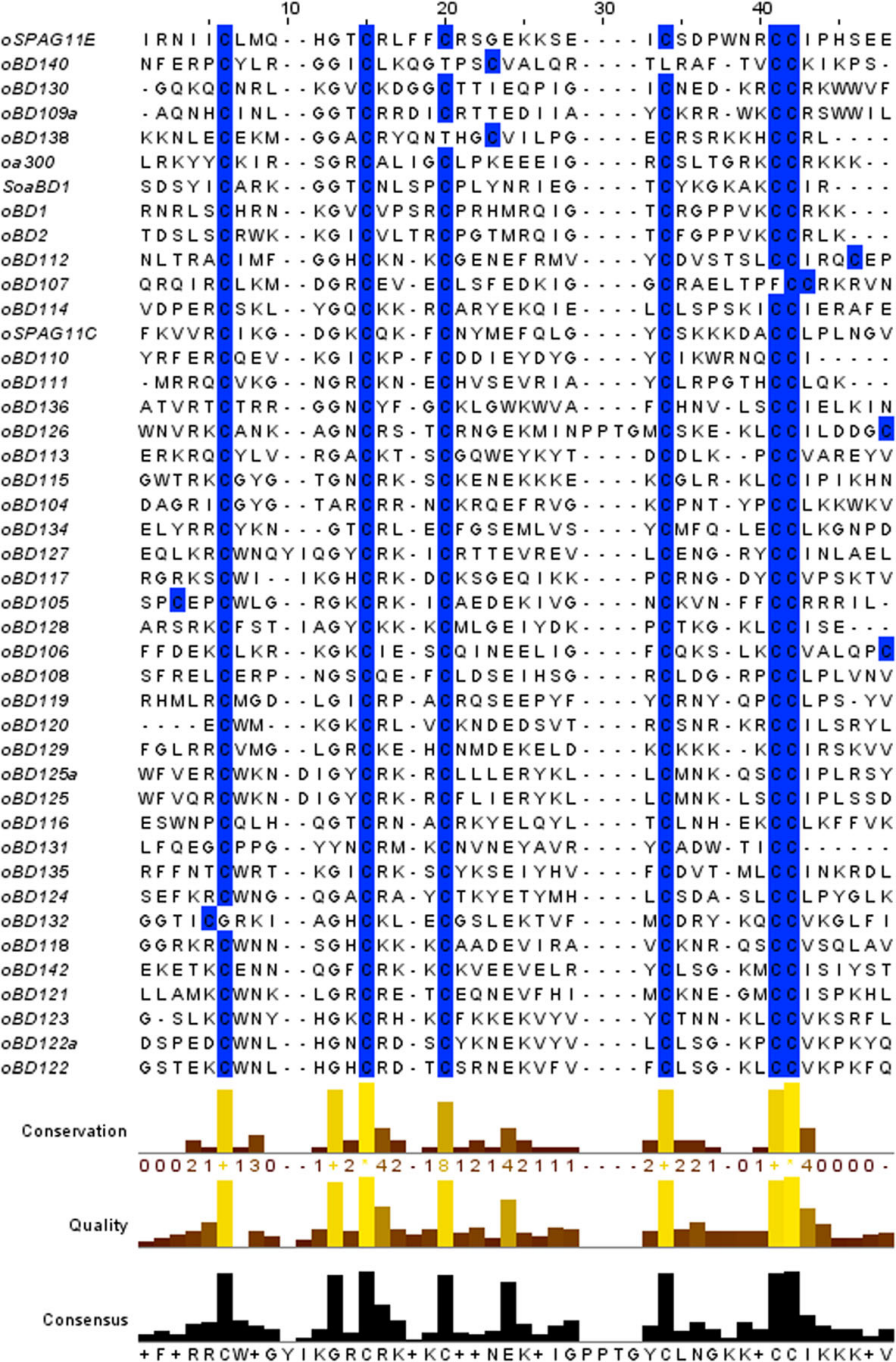
Jalview multiple sequence alignment of the second exons of the 43 ovine β-defensins highlighting the conserved cysteine residues (shaded) characteristic of β-defensin genes. The level of amino acid conservation is shown at the bottom of the figure

On the basis of these filtered hits, all 41 putative novel ovine β-defensin genes plus two previously characterised ovine β-defensins (oBD1 and oBD2) were aligned (Fig. 1). The characteristic 6 cysteine signature was conserved in all β-defensins, although the spacing between cysteines varied. The cysteines represent the only sites at which the amino acid sequences are 100% conserved, with significant diversity between amino acid residues constituting the remainder of exon 2.

oBD140 has only 5 cysteines in its sequence, which differs from the usual 6 cysteine defensin motif. oBD140, shares high similarity with bBD140 between their first exon and the first half of the second exon. However, the second half of the second exon differs greatly from any other defensin gene. Upon further analysis, it was discovered that a thymine base is missing from the reference genome, causing a frameshift leading to the generation of an incomplete second exon and thus a vastly different mature peptide. Whether this is an assembly issue or a prevalent mutation in the population remains unclear.

In contrast oBD112, oBD106 and oBD126 all contain an additional cysteine, all of which occur after the 6^th^ canonical one (Fig. 1).

### Analysis of ovine and bovine β-defensin phylogeny and gene order conservation – 4 distinct genomic clusters

**Fig. 2:**
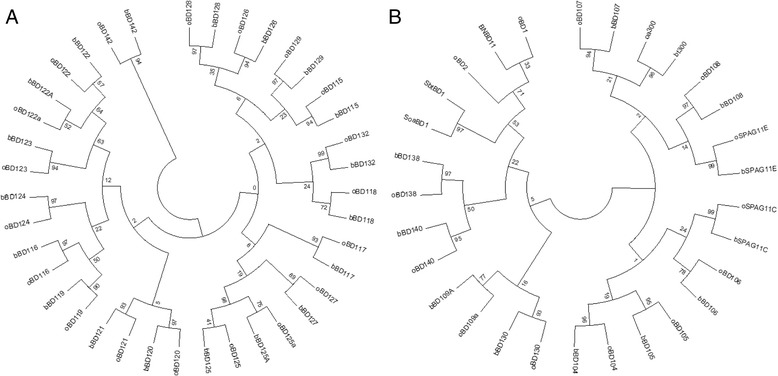
Maximum likelihood phylogenetic tree for ovine β-defensin proteins compared to their bovine orthologs on (**a**) chromosome 13 and (**b**) chromosome 26, respectively. Trees were constructed using full length β-defensin sequences from *Bos taurus* and *Ovis aries* aligned via Clustal omega, representing two of four β-defensin clusters. The values at each node represent percentage bootstrap scores at 1000 iterations

Upon completion of the first and second exon annotation of the 41 newly characterised ovine β-defensins, a phylogenetic analysis was carried out using MEGA 5.1 to determine the phylogeny between the bovine and ovine β-defensin sequences. Newly annotated ovine β-defensins were named based on their sequence similarity with the bovine β-defensin with which it was found. Results indicate a strong evolutionary relationship between the bovine and ovine genes. However, due to the short sequence length, high phylogenetic support can usually only be determined between homologs of high similarity (Fig. 2a). Clear 1:1 orthologs of ovine *BD1* and *BD2* are not present in the bovine genome but both showed a uniform similarity with the 11 member BNBD gene family in *Bos taurus*, and had 1% greater similarity with BNBD11, as indicated by the phylogenetic tree (Fig. 2b).

**Fig. 3:**
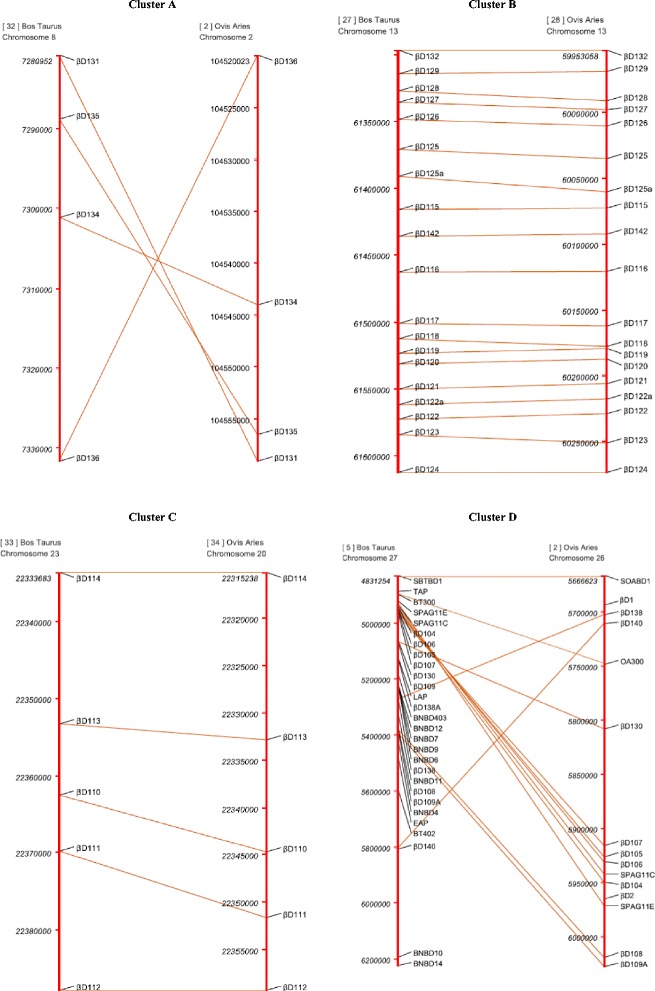
Gene order maps of the four β-defensin clusters found in *Bos taurus* and *Ovis aries*, respectively. Clusters are denoted *A* (OA chromosome 2/ BT chromosome 8); *B* (OA chromosome 13/ BT chromosome 13); *C* (OA chromosome 20/ BT chromosome 23) and *D* (OA chromosome 26/ BT chromosome 27). Genes without connecting vectors have no match within the other species

The gene order conservation of β-defensin gene clusters between bovine and ovine β-defensins was investigated and the chromosomal arrangement or overall defensin orientation of these genes agreed with what we previously published in cattle [42]. Four distinct clusters were identified, and to ensure consistency for comparison to closely related species were named Clusters A to D. The similarity in gene orientation of ovine chromosome 2 is highly similar to bovine chromosome 8 with the exception of a possible inversion in gene order (Fig. 3a). Bovine chromosome 13 shares 1:1 gene orientation with sheep chromosome 13 (Fig. 3b), as does chromosome 20 in sheep with chromosome 23 in cattle (Fig. 3c). In contrast, the conservation of gene order between ovine chromosome 26 and bovine chromosome 27 is very low, with many genes missing from the ovine chromosome 26 repertoires (Fig. 3d).

Cluster A consists of 4 genes located on Chromosome 2 in sheep and 8 in cattle (*oBD136*, *oBD134*, *oBD135* and *oBD131*). Cluster B consists of 19 genes on chromosome 13 in sheep (*oBD132*, *oBD129*, *oBD128, oBD127, oBD126, oBD125, oBD125a, oBD115, oBD142, oBD116, oBD117, oBD118, oBD119, oBD120, oBD121, oBD122a, oBD122, oBD123* and *oBD124*). Cluster C consists of 5 genes on chromosome 20 (*oBD114, oBD113, oBD110, oBD111* and *oBD112*); and finally, cluster D consists of 15 genes on chromosome 26 (*SoABD1, oBD1, oBD138, oBD140, Def300, oBD130, oBD107, oBD105, oBD106, SPAG11C, oBD104, oBD2, SPAG11E, oBD108* and *oBD109a*).

Sequence matches for bovine *TAP, bBD109, BNBD403, BNBD6, BNBD7, BNBD8, BNBD9, BNBD11, BNBD12, BNBD4, BNBD14 BNBD10, EAP, LAP* and *BT402* could not be conclusively identified in the sheep genome, as each of these genes matched sheep β-defensin 1 and 2 with almost uniform similarity (67-69%). Of the 41 new ovine genes identified, 16 had been provisionally characterised at the computational level previously (*oBD127, oBD116, oBD110, oBD125, oBD119, oBD118, oBD105, oBD134, oBD112, oBD115, oBD129, oBD112, oBD108, oBD123, oBD124* and *SPAG11*). As indicated in Fig. 3, there is a high level of conservation in gene order between 3 of the four clusters. A clear feature of this conservation is that the defensin clusters span similar chromosomal distances between cattle and sheep. Bovine β-defensin cluster on chromosome 13 spans 315,458 nucleotide bases. Similarly, the sheep cluster on chromosome 13 spans 296,984 bases. Likewise, there is only a difference of 7,017 bases between cattle chromosome 23 and sheep 20 and a difference of 1,588 bases between chromosome 8 and 2. In contrast, bovine β-defensin cluster on chromosome 27 spans 584,753 bases, whereas, the ovine cluster on chromosome 26 spans only 346,101 bases.

### Expression of novel β-defensins in reproductive tissues of the ram

**Fig. 4:**
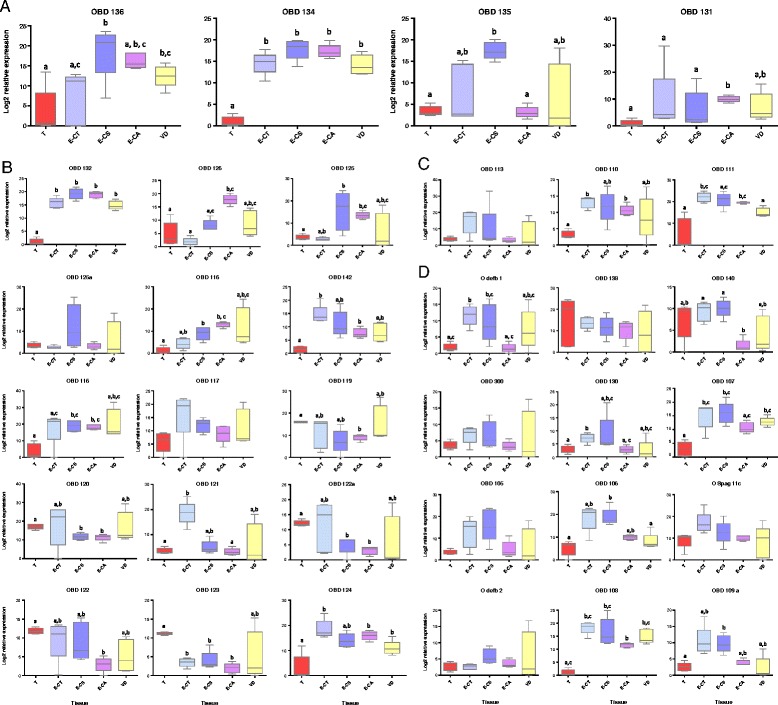
Relative gene expression profiles for β-defensin genes across reproductive tissues of the ram (*n* = 5) and clustered by chromosomal location. **a** genes on ovine Chromosome 2; (**b**) genes on ovine Chromosome 13; (**c**) genes on ovine Chromosome 20 and (**d**) genes on ovine Chromosome 26. Tissues are identified on the x-axis; T-testes; E-CT, caput epididymis; E-CS, corpus epididymus; E-CA, cauda epididymis and VD, vas deferens. Significant differences are denoted with different subscripts above each box-plot (*P* < 0.01)

The expression profiles of 34 β-defensin genes, which included the previously examined *oBD1* and *oBD2*, were examined across the five distinct tissues (testes (T), caput (E-CT), corpus (E-CS) and cauda (E-CA) epididymis as well as the vas deferens (VD)) from mature rams (Fig. 4). The genes were chosen to represent each of the four gene clusters (denoted A-D) and corresponding to chromosomes 2, 13, 20 and 26 in sheep, respectively.

Cluster A –Differential expression was detected between tissue sections for all four genes (Fig. 4(a); *P* < 0.01). For each gene in the cluster, lowest gene expression was detected in the testes, and maximal gene expression in the epididymis. *oBD136* showed significantly elevated expression in the corpus epididymis as well as the vas deferens relative to the expression level in the testes. Expression of *oBD134* was significantly elevated in all epididymal regions and vas deferens compared to the testes. Similarly, *oBD135* expression was significantly higher in E-CS than in the testes. A significant increase in *oBD131* expression was detected in the cauda, relative to the testes.

Cluster B –Differential expression was detected between tissue sections for 13 out of the 15 genes characterised by qPCR in this cluster (Fig. 4(b); *P* < 0.01). Interestingly, whereas *oBD125* was significantly differentially expressed, its closely related counterpart *oBD125a* was not. Maximal gene expression levels were detected in the testes for only three genes – *oBD119*, *oBD122* and *oBD123. oBD119* and *oBD122* expression was significantly lower in the E-CL, and *oBD123* exhibited significantly lower expression in all three epididymal regions relative to the testes. Maximal gene expression levels were detected in the E-CT for *oBD142*, *oBD116*, *oBD117*, *oBD120*, *oBD121*, *oBD122a* and *oBD124*. Maximal gene expression levels were detected in the corpus epididymis for only *oBD132*, *oBD125* and *oBD125a*. Only *oBD126* and *oBD115* showed maximal gene expression in the cauda epididymis. No gene exhibited maximal expression in the vas deferens. In general, inter-ram gene expression levels were found to be most variable in the vas deferens.

Cluster C – Two of the three genes characterised in this cluster showed significant differential expression between tissue sections (*oBD110* and *oBD111*, Fig. 4(c); *P* < 0.01). In both cases, gene expression was lowest in the testes and significantly elevated in all epididymal regions. Gene expression for *oBD111* was significantly reduced in the vas deferens .

Cluster D – Twelve genes in this cluster were assessed and differential expression was detected between tissue sections for seven genes (Fig. 4(d); *P* < 0.01). This cluster contains the two already characterised ovine β-defensins, *oBD1* and *oBD2*. Whereas *oBD1* was significantly elevated in the caput and the corpus epididymis compared to the testes, *oBD2* was not differentially expressed. All significantly differentially expressed genes (*oBD1*, *oBD140*, *oBD130*, *oBD107*, *oBD106*, *oBD108* and *oBD109a*) exhibited maximal expression in the caput. A general trend of reduced expression was apparent for these genes in the cauda epididymis and vas deferens.

Of the 34 genes profiled by qPCR, 26 were found to be significantly differentially expressed across reproductive tissues of the ram. No clear differences in expression profile between chromosomal clusters were observed.

## Discussion

Although defensins have been extensively characterised in humans, rodents, chickens and cattle, β-defensin families remain uncharacterised in many non-model organisms. Identifying novel AMPs in diverse species has significant potential for understanding the evolutionary history of this important gene family and also for application in breeding programmes for multiple livestock species [42]. The increasing availability of genomic sequence data via advances in next-generation sequencing facilitates a more comprehensive assessment of the β-defensin gene complement across species. A comparative genomics approach has previously been used to identify new gene families in chickens, cattle and horses [20, 29, 43]. Species specific differences in β-defensin gene number suggest that multiple gene-duplication events and sequence diversification in the lineage leading to mammals has resulted in many β-defensins with divergent amino acid composition but almost identical tertiary structures, even amongst closely related species [44]. This could possibly be due to the specific pathogenic niche-based insults members of closely related species are exposed to over the course of their evolution [45].

The identification of 41 novel β-defensin genes in the *Ovis aries* genome here brings the current tally of β-defensin genes characterised to 43, but the final number is likely to change in line with increasingly accurate annotation of the ovine reference genome. The comparison of the 3 bovine β-defensin clusters which share a high level of gene order conservation with the ovine β-defensin clusters in this study indicates common ancestry for these genes, as well as positive selective pressure to maintain these genes through the divergence of the different ruminant species. Comparison of the bovine chromosome 27 cluster to the ovine chromosome 26 cluster reveals an expansion of bovine β-defensins not seen in *Ovis aries* illustrating the discontinuous nature of the β-defensin catalogue in mammals, where in some cases individual genes can be gained or lost, perhaps due to varying niche-based pathogenic insults. Future more accurate annotation of livestock genomes will likely shed light on the specific genomic changes in β-defensin gene repertoire between species.

Bovinae and Caprinae (which includes sheep) diverged about 20 million years ago [46] and a plausible explanation for the selection pressure which originally drove the expansion of large β-defensin gene families was postulated to be the evolution of the rumen (For review see: [42]). It is thought that the complexity of the microbial populations within the rumen required a vast array of defence peptides to manage the mucosal interface and prevent dissemination resulting in inflammation and disease. However, the presence of these genes in monogastrics including horses [29] and pigs [47] supports an alternative rationale. While the retention of these genes in monogastrics does not exclude a role in managing the mucosal microflora, it is likely that defensins play a more widespread and direct role in immunoprotection across body surfaces in mammals.

Only two β-defensin genes have previously been characterised in sheep (*oBD1* and *oBD2*), and their expression documented in mucosal tissue in the trachea and ileum [24, 25]. However, no previous study examined expression of these or related sequences in reproductive tissues in sheep. Here, we demonstrate extensive but site-specific expression of an expanded repertoire of 43 β-defensin genes in the reproductive tract of the ram. In agreement with our previous results in cattle, expression of all of these genes is highest in the epididymis and particularly in the caudal region [21, 22], suggesting a role for these molecules in sperm function and fertility.

A remarkable recent discovery has been the identification of a seminal fluid microbiome in mice [48]. It is logical, given the abundance of amino acids and other nutritional substrates in basic seminal fluid, that it would promote bacterial growth. In fact, researchers now propose that this microbiome impacts directly on the etiology of infertility [49]. In this context, it now seems plausible to propose that β-defensins evolved to regulate the microbiome in seminal fluid and prevent the growth of bacterial populations that may be detrimental to either sperm quality or uterine health. However, detailed follow on studies are required to further investigate this hypothesis. The expression of all evaluated β-defensins in the reproductive tract of the ram (and similarly in other species) shown here highlights the priority to which prevention of ascending infection acquired over the course of evolution. It is relevant that infectious epididymitis is considered a major cause of economic losses for the sheep industry worldwide [50, 51] and future work should examine the potential efficacy of epididymal β-defensins against the causative bacteria.

## Conclusions

The high level of gene expression and region-specific changes in β-defensin gene expression suggest epididymal specific roles for these molecules during sperm maturation in sheep, in line with what has been documented in other species. This bioinformatics search and validation has brought the current number of β-defensin genes in sheep to 43. Increasing our knowledge of the structure and composition of AMP related genes sheds light on the divergent evolution of the immune system of different ruminants, as well as identifying new targets to breed for traits of agricultural and economic importance.

## Additional file

Additional file 1: A. Gene and Protein sequences for ovine β-defensin genes. B. Oligonucleotide primers for ovine β-defensin genes. (XLSX 21 kb)

## Abbreviations

bBD: bovine beta-defensin
BLAST: basic local alignment search tool
BLAT: blast-like alignment tool
HMM: hidden markov models
NCBI: national center for biotechnology information
oBD: ovine beta-defensin

## References

[1] Ganz T (2004). Defensins: antimicrobial peptides of vertebrates. C R Biol.

[2] Bruhn O, Paul S, Tetens J, Thaller G (2009). The repertoire of equine intestinal alpha-defensins. BMC Genomics.

[3] Tang YQ, Yuan J, Osapay G, Osapay K, Tran D, Miller CJ, Ouellette AJ, Selsted ME (1999). A cyclic antimicrobial peptide produced in primate leukocytes by the ligation of two truncated alpha-defensins. Science.

[4] Ganz T (2003). Defensins: antimicrobial peptides of innate immunity. Nat Rev Immunol.

[5] Schneider JJ, Unholzer A, Schaller M, Schafer-Korting M, Korting HC (2005). Human defensins. J Mol Med (Berl).

[6] Ganz T (1999). Defensins and host defense. Science.

[7] Ganz T, Selsted ME, Szklarek D, Harwig SS, Daher K, Bainton DF, Lehrer RI (1985). Defensins. Natural peptide antibiotics of human neutrophils. J Clin Investig.

[8] Bowdish DM, Davidson DJ, Scott MG, Hancock RE (2005). Immunomodulatory activities of small host defense peptides. Antimicrob Agents Chemother.

[9] Bowdish DM, Davidson DJ, Hancock RE (2005). A re-evaluation of the role of host defence peptides in mammalian immunity. Curr Protein Pept Sci.

[10] Yang D, Chertov O, Bykovskaia SN, Chen Q, Buffo MJ, Shogan J, Anderson M, Schroder JM, Wang JM, Howard OM (1999). Beta-defensins: linking innate and adaptive immunity through dendritic and T cell CCR6. Science.

[11] Dorin JR, Barratt CL (2014). Importance of beta-defensins in sperm function. Mol Hum Reprod.

[12] Thimon V, Koukoui O, Calvo E, Sullivan R (2007). Region-specific gene expression profiling along the human epididymis. Mol Hum Reprod.

[13] Belleannee C, Thimon V, Sullivan R (2012). Region-specific gene expression in the epididymis. Cell Tissue Res.

[14] Browne JA, Yang R, Leir SH, Eggener SE, Harris A (2016). Expression profiles of human epididymis epithelial cells reveal the functional diversity of caput, corpus and cauda regions. Mol Hum Reprod.

[15] Tollner TL, Yudin AI, Treece CA, Overstreet JW, Cherr GN (2008). Macaque sperm coating protein DEFB126 facilitates sperm penetration of cervical mucus. Hum Reprod.

[16] Tollner TL, Yudin AI, Treece CA, Overstreet JW, Cherr GN (2004). Macaque sperm release ESP13.2 and PSP94 during capacitation: the absence of ESP13.2 is linked to sperm-zona recognition and binding. Mol Reprod Dev.

[17] Tollner TL, Yudin AI, Tarantal AF, Treece CA, Overstreet JW, Cherr GN (2008). Beta-defensin 126 on the surface of macaque sperm mediates attachment of sperm to oviductal epithelia. Biol Reprod.

[18] Tollner TL, Venners SA, Hollox EJ, Yudin AI, Liu X, Tang G, Xing H, Kays RJ, Lau T, Overstreet JW (2011). A common mutation in the defensin DEFB126 causes impaired sperm function and subfertility. Sci Transl Med.

[19] Zhao Y, Diao H, Ni Z, Hu S, Yu H, Zhang Y (2011). The epididymis-specific antimicrobial peptide beta-defensin 15 is required for sperm motility and male fertility in the rat (Rattus norvegicus). Cell Mol Life Sci.

[20] Cormican P, Meade KG, Cahalane S, Narciandi F, Chapwanya A, Lloyd AT, O'Farrelly C (2008). Evolution, expression and effectiveness in a cluster of novel bovine beta-defensins. Immunogenetics.

[21] Narciandi F, Lloyd AT, Chapwanya A, O' Farrelly C, Meade KG (2011). Reproductive tissue-specific expression profiling and genetic variation across a 19 gene bovine beta-defensin cluster. Immunogenetics.

[22] Narciandi F, Fernandez-Fuertes B, Khairulzaman I, Jahns H, King D, Finlay EK, Mok KH, Fair S, Lonergan P, O'Farrelly C, et al. Sperm-Coating Beta-Defensin 126 Is a Dissociation-Resistant Dimer Produced by Epididymal Epithelium in the Bovine Reproductive Tract. Biol Reprod. 2016. 10.1095/biolreprod.116.138719 PMC5333941 27707712

[23] Fernandez-Fuertes B, Narciandi F, O'Farrelly C, Kelly AK, Fair S, Meade KG, Lonergan P. Cauda Epididymis-Specific Beta-Defensin 126 Promotes Sperm Motility but Not Fertilizing Ability in Cattle. Biol Reprod. 2016. 10.1095/biolreprod.116.138792 PMC5333942 27707713

[24] Huttner KM, Lambeth MR, Burkin HR, Burkin DJ, Broad TE (1998). Localization and genomic organization of sheep antimicrobial peptide genes. Gene.

[25] Huttner KM, Brezinski-Caliguri DJ, Mahoney MM, Diamond G (1998). Antimicrobial peptide expression is developmentally regulated in the ovine gastrointestinal tract. J Nutr.

[26] Meyerholz DK, Gallup JM, Grubor BM, Evans RB, Tack BF, McCray PB, Ackermann MR (2004). Developmental expression and distribution of sheep beta-defensin-2. Dev Comp Immunol.

[27] Grubor B, Gallup JM, Meyerholz DK, Crouch EC, Evans RB, Brogden KA, Lehmkuhl HD, Ackermann MR (2004). Enhanced surfactant protein and defensin mRNA levels and reduced viral replication during parainfluenza virus type 3 pneumonia in neonatal lambs. Clin Diagn Lab Immunol.

[28] Ackermann MR, Gallup JM, Zabner J, Evans RB, Brockus CW, Meyerholz DK, Grubor B, Brogden KA (2004). Differential expression of sheep beta-defensin-1 and -2 and interleukin 8 during acute Mannheimia haemolytica pneumonia. Microb Pathog.

[29] Johnson GP, Lloyd AT, O'Farrelly C, Meade KG, Fair S. Comparative genomic identification and expression profiling of a novel B-defensin gene cluster in the equine reproductive tract. Reprod Fertil Dev. 2015. 10.1071/RD14345 25924226

[30] Altschul SF, Gish W, Miller W, Myers EW, Lipman DJ (1990). Basic local alignment search tool. J Mol Biol.

[31] Sievers F, Higgins DG (2014). Clustal Omega, accurate alignment of very large numbers of sequences. Methods Mol Biol.

[32] Waterhouse AM, Procter JB, Martin DM, Clamp M, Barton GJ (2009). Jalview Version 2--a multiple sequence alignment editor and analysis workbench. Bioinformatics.

[33] Kent WJ (2002). BLAT--the BLAST-like alignment tool. Genome Res.

[34] Korf I, Flicek P, Duan D, Brent MR (2001). Integrating genomic homology into gene structure prediction. Bioinformatics.

[35] Rice P, Longden I, Bleasby A (2000). EMBOSS: the European Molecular Biology Open Software Suite. Trends Genet.

[36] Eddy SR (2011). Accelerated Profile HMM Searches. PLoS Comput Biol.

[37] Notredame C, Higgins DG, Heringa J (2000). T-Coffee: A novel method for fast and accurate multiple sequence alignment. J Mol Biol.

[38] Hall BG (2013). Building phylogenetic trees from molecular data with MEGA. Mol Biol Evol.

[39] Paterson T, Law A (2013). ArkMAP: integrating genomic maps across species and data sources. BMC Bioinformatics.

[40] Vandesompele J, De Preter K, Pattyn F, Poppe B, Van Roy N, De Paepe A, Speleman F. Accurate normalization of real-time quantitative RT-PCR data by geometric averaging of multiple internal control genes. Genome Biol. 2002;3(7):RESEARCH0034. 10.1186/gb-2002-3-7-research0034 PMC126239 12184808

[41] Livak KJ, Schmittgen TD (2001). Analysis of relative gene expression data using real-time quantitative PCR and the 2(-Delta Delta C(T)) Method. Methods.

[42] Meade KG, Cormican P, Narciandi F, Lloyd A, O'Farrelly C (2014). Bovine beta-defensin gene family: opportunities to improve animal health?. Physiol Genomics.

[43] Higgs R, Lynn DJ, Gaines S, McMahon J, Tierney J, James T, Lloyd AT, Mulcahy G, O'Farrelly C (2005). The synthetic form of a novel chicken beta-defensin identified in silico is predominantly active against intestinal pathogens. Immunogenetics.

[44] Bauer F, Schweimer K, Kluver E, Conejo-Garcia JR, Forssmann WG, Rosch P, Adermann K, Sticht H (2001). Structure determination of human and murine beta-defensins reveals structural conservation in the absence of significant sequence similarity. Protein Sci.

[45] Radhakrishnan Y, Hamil KG, Yenugu S, Young SL, French FS, Hall SH (2005). Identification, characterization, and evolution of a primate beta-defensin gene cluster. Genes Immun.

[46] Kumar S, Hedges SB (1998). A molecular timescale for vertebrate evolution. Nature.

[47] Guyonnet B, Marot G, Dacheux JL, Mercat MJ, Schwob S, Jaffrezic F, Gatti JL (2009). The adult boar testicular and epididymal transcriptomes. BMC Genomics.

[48] Javurek AB, Spollen WG, Ali AM, Johnson SA, Lubahn DB, Bivens NJ, Bromert KH, Ellersieck MR, Givan SA, Rosenfeld CS (2016). Discovery of a Novel Seminal Fluid Microbiome and Influence of Estrogen Receptor Alpha Genetic Status. Scientific reports.

[49] Weng SL, Chiu CM, Lin FM, Huang WC, Liang C, Yang T, Yang TL, Liu CY, Wu WY, Chang YA (2014). Bacterial communities in semen from men of infertile couples: metagenomic sequencing reveals relationships of seminal microbiota to semen quality. PloS One.

[50] Moustacas VS, Silva TM, Costa LF, Carvalho Junior CA, Santos RL, Paixao TA (2014). Clinical and pathological changes in rams experimentally infected with Actinobacillus seminis and Histophilus somni. TheScientificWorldJournal.

[51] DeLong WJ, Waldhalm DG, Hall RF (1979). Bacterial isolates associated with epididymitis in rams from Idaho and eastern Oregon flocks. Am J Vet Res.

